# Supplementary material for: Superporous sponge prepared by secondary network compaction with enhanced permeability and mechanical properties for non-compressible hemostasis in pigs
Source: Nat Commun. 2024 Jun 27;15:5460. doi: 10.1038/s41467-024-49578-2 (PMC11211411; doi:10.1038/s41467-024-49578-2)
Supplement: Supplementary file 1 — Supplementary information [file 41467_2024_49578_MOESM1_ESM.pdf]

Supplementary information for

Superporous sponge prepared by secondary network compaction with enhanced permeability and mechanical properties for non-compressible hemostasis in pigs

Tianshen Jiang[1,2], Sirong Chen[1,2], Jingwen Xu[1,2], Yuxiao Zhang[1,2], Hao Fu[1,2], Qiangjun Ling[1,2], Yan Xu[3], Xiangyu Chu[4], Ruinan Wang[1,2], Liangcong Hu[4], Hao Li[5], Weitong Huang[1,2], Liming Bian[1,2,6,7]*, Pengchao Zhao[1,2,6,7]*, Fuxin Wei[3,8]*

[1]School of Biomedical Sciences and Engineering, Guangzhou International Campus, South China University of Technology, Guangzhou, 511442, China
[2]National Engineering Research Center for Tissue Restoration and Reconstruction, South China University of Technology, Guangzhou, 510006, China
[3]Department of Orthopedic Surgery, The Seventh Affiliated Hospital of Sun Yat-sen University, Shenzhen, 518107 P. R. China
[4]Department of Orthopedics, Union Hospital, Tongji Medical College, Huazhong University of Science and Technology, Wuhan, 430022, China
[5]Department of Joint Surgery, First Affiliated Hospital of Sun Yat-sen University, Guangzhou, 510080, China
[6]Guangdong Provincial Key Laboratory of Biomedical Engineering, South China University of Technology, Guangzhou 510006, China
[7]Key Laboratory of Biomedical Materials and Engineering of the Ministry of Education, South China University of Technology, Guangzhou 510006, China
[8]Shenzhen Key Laboratory of Bone Tissue Repair and Translational Research, Shenzhen, 518107 P. R. China

These authors contributed equally to this work:
Tianshen Jiang, Sirong Chen, Jingwen Xu

*Corresponding authors. Emails: bianlm@scut.edu.cn (L.B.); scutzpc1993@scut.edu.cn (P.Z.); weifuxin@mail.sysu.edu.cn (F.W.)

Supplementary Figures

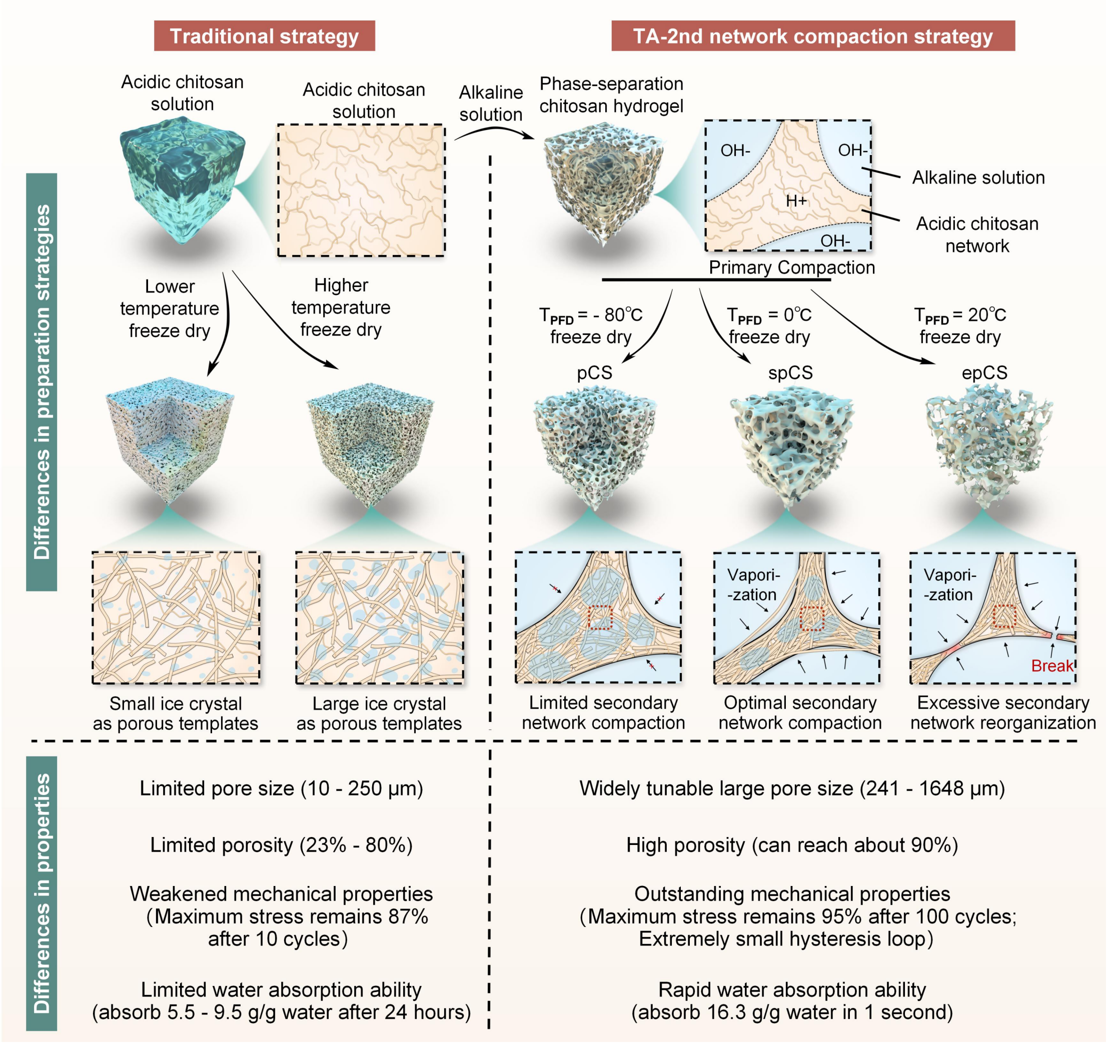

Figure S1. Preparation strategies differences and properties differences between traditional strategy and temperature-assisted secondary network compaction (TA-2nd NC) strategy.

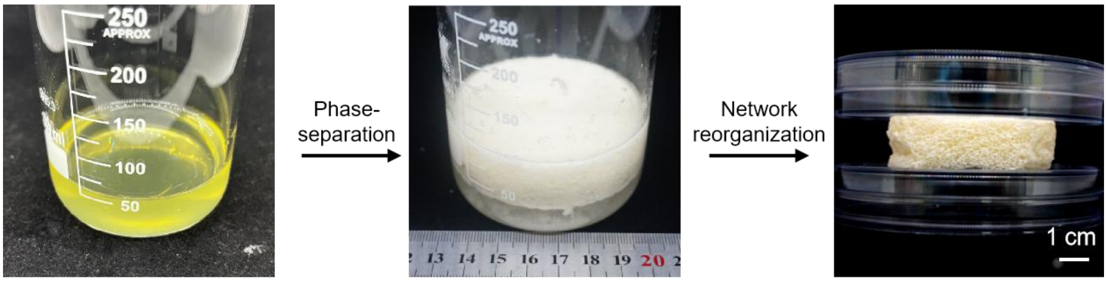

Figure S2. Photographs of the fabrication process of spCS. Sodium bicarbonate solution was added to acidic chitosan solution to induce phase separation. The superporous chitosan sponge (spCS) was obtained temperature-assisted secondary network compaction.

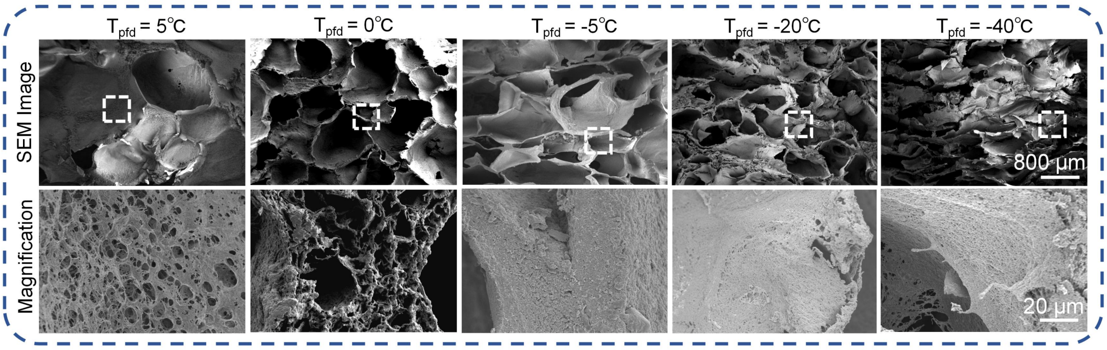

Figure S3. Scanning electron microscope (SEM) images of the microstructure of sponges prepared at pre-freeze drying temperatures (T_{pfd}) of 5, -5, -20, 0, and -40℃.

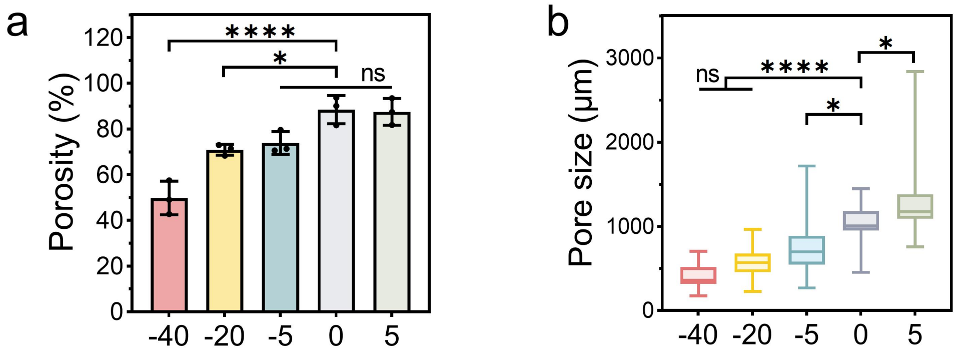

Figure S4. Porosity and pore size of sponges prepared at pre-freeze drying temperatures of 5, -5, -20, 0, and -40℃. **a)** Porosity of sponges prepared at T_{pfd} of -40, -20, -5, 0, and 5℃. **b)** Pore size of sponges prepared at T_{pfd} of -40, -20, -5, 0, and 5℃ (n=25). Values and error bars in (b) represent the mean and standard deviation, P > 0.05 (ns), **P < 0.01, ***P < 0.001, ****P < 0.0001 (one-way ANOVA to compare multiple groups or two groups).

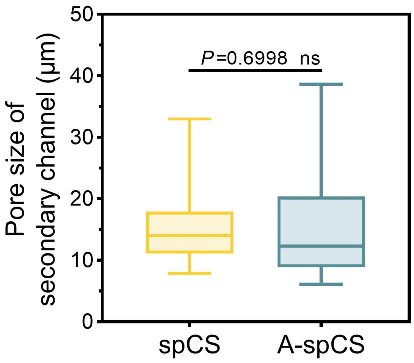

Figure S5. Pore diameter of secondary channel spCS and A-spCS (n=25). Values and error bars represent the mean and standard deviation, P > 0.05 (ns), **P < 0.01, ***P < 0.001, ****P < 0.0001 (Two-tailed Student's t-test to compare two groups).

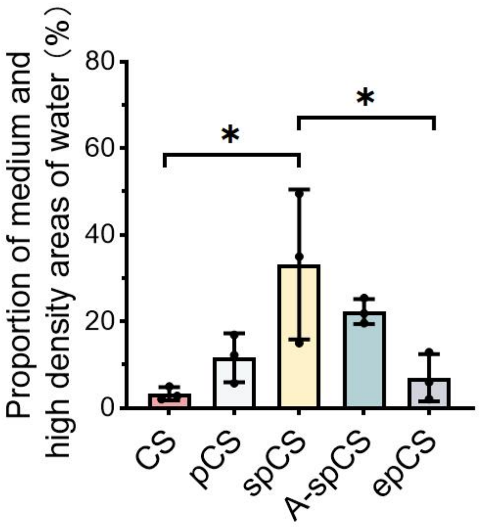

Figure S6. Proportion of medium-density and high-density areas in CS, pCS, spCS, A-spCS, and epCS. Values and error bars represent the mean and standard deviation (n = 3 independent samples), P > 0.05 (ns), **P < 0.01, ***P < 0.001, ****P < 0.0001 (one-way ANOVA to compare multiple groups or two groups).

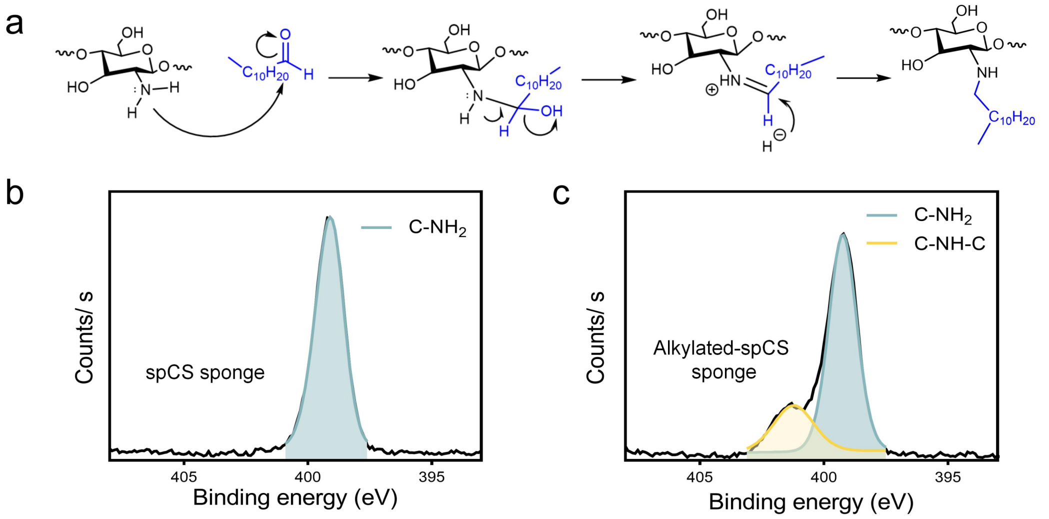

Figure S7. spCS grafted with alkyl chains by Schiff base reaction and reduced by triethoxyborohydride. **a)** Schematic diagram of alkylation modification reaction mechanism. X-ray photoelectron spectroscopy (XPS) spectra of spCS **b)** and A-spCS **c)** with N1*s* peak.

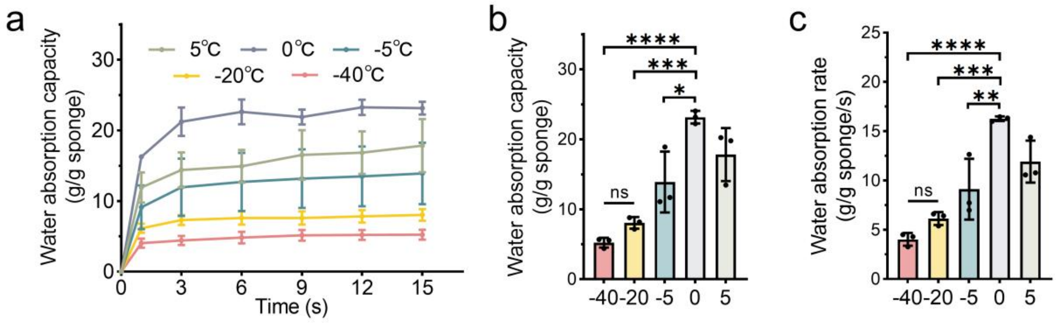

Figure S8. Water absorption ability of sponges prepared at T_{pfd} of -40, -20, -5, 0, and 5°C. **a)** Water absorption curve of sponges prepared at T_{pfd} of -40, -20, -5, 0, and 5°C in 15 seconds. **b)** Water absorption capacity of sponges prepared at T_{pfd} of -40, -20, -5, 0, and 5°C. **c)** Water absorption rate of sponges prepared at T_{pfd} of -40, -20, -5, 0, and 5°C. Values and error bars represent the mean and standard deviation (n = 3 independent samples), P > 0.05 (ns), **P < 0.01, ***P < 0.001, ****P < 0.0001 (one-way ANOVA to compare multiple groups or two groups).

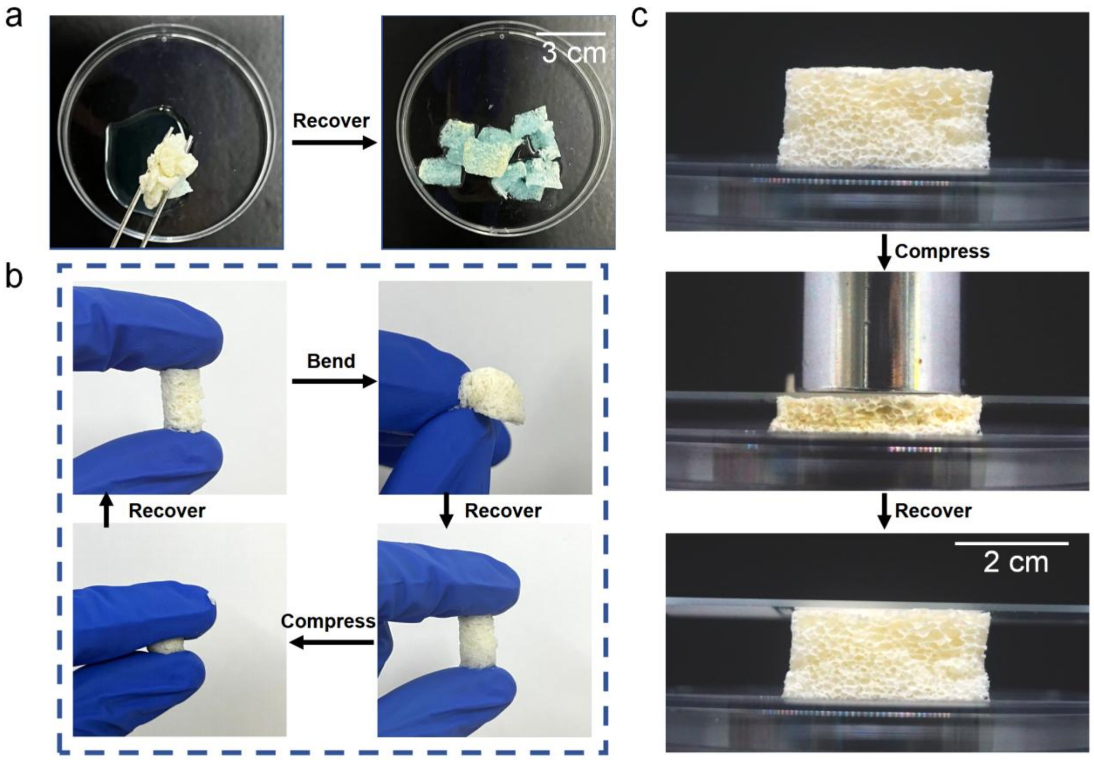

Figure S9. Deformation, compression, and shape recovery of A-spCS. **a)** The compressed chitosan sponge absorbs a solution containing methylene blue for rapid shape recovery. **b)** Macroscopic morphology changes of chitosan sponge during bending, compression, and recovery. **c)** spCS achieves shape recovery after weight compression.

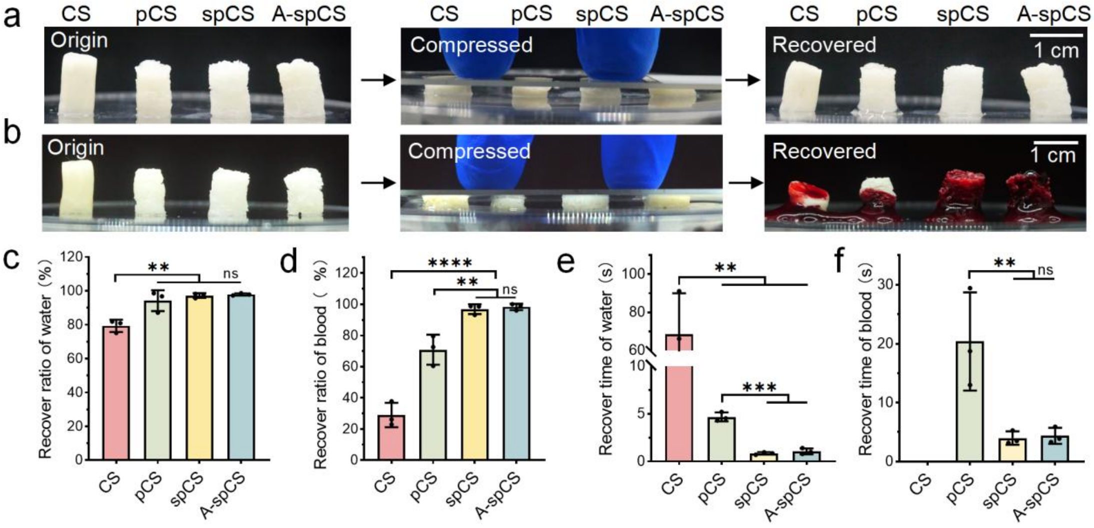

Figure S10. Chitosan sponge shape recovery ratio and rate statistics. **a, b)** Macrophotograph of CS, pCS, spCS, A-spCS absorbing water-triggered/blood-triggered shape recovery. **c)** Volume recovery ratio of CS, pCS, spCS, and A-spCS after water absorption. **d)** Volume recovery ratio of CS, pCS, spCS, and A-spCS after blood absorption. **e)** Total recovery time of CS, pCS, spCS, and A-spCS after water absorption. **f)** Total recovery time of CS, pCS, spCS, and A-spCS after blood absorption. Values and error bars represent the mean and standard deviation (n = 3 independent samples), P > 0.05 (ns), **P < 0.01, ***P < 0.001, ****P < 0.0001 (one-way ANOVA or Two-tailed Student's t-test to compare multiple groups or two groups).

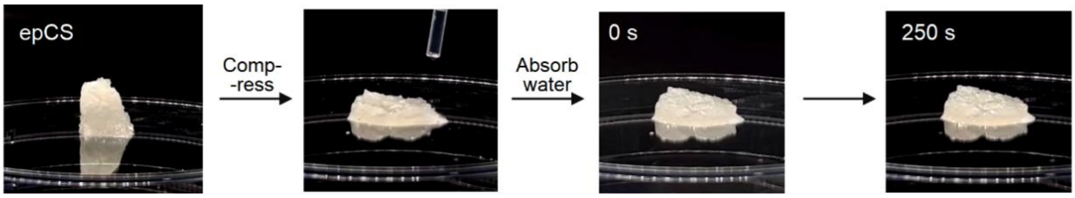

Figure S11. epCS suffered irreversible structural damage after compression, resulting in the inability to recover its shape after absorbing water.

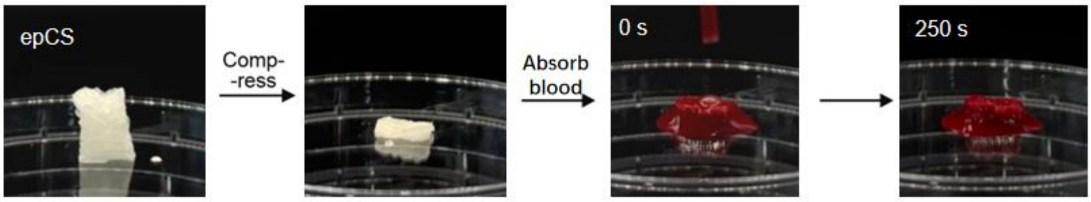

Figure S12. epCS suffered irreversible structural damage after compression, resulting in the inability to recover its shape after absorbing blood.

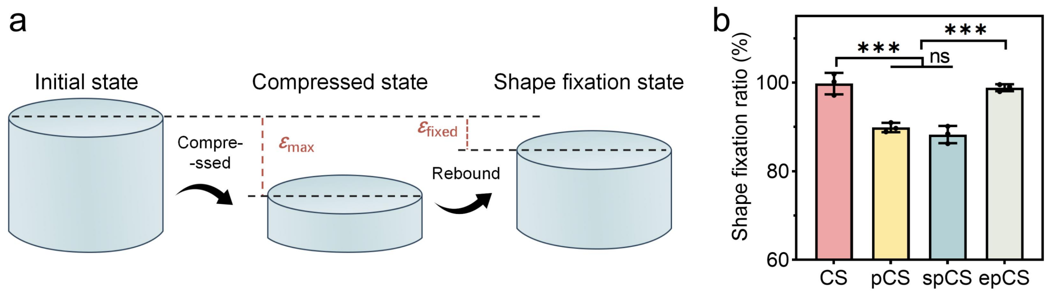

Figure S13. Shape fixation ratio of CS, pCS, spCS, and epCS. **a**) A schematic diagram illustrating the compressed state and shape fixation state. **b**) Statistical graph depicting the shape fixation ratio. Values and error bars represent the mean and standard deviation (n = 3 independent samples), P > 0.05 (ns), **P < 0.01, ***P < 0.001, ****P < 0.0001 (one-way ANOVA to compare multiple groups or two groups).

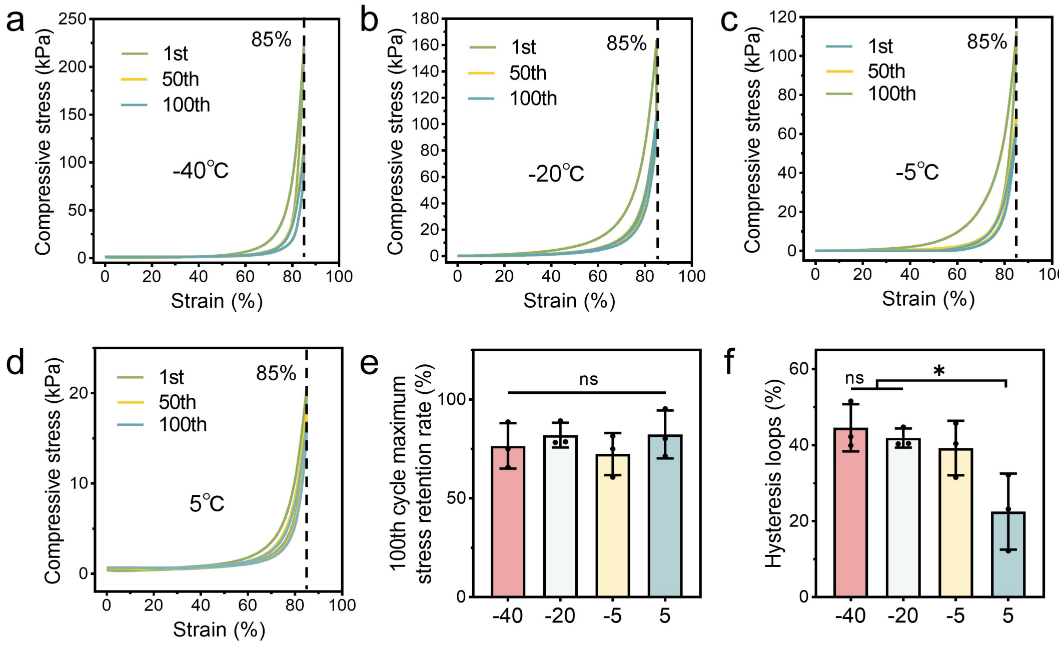

Figure S14. Mechanical performance test of sponges prepared at T_{pfd} of -40, -20, -5, and 5°C. **a-d)** Stress-strain cyclic curves sponges prepared at T_{pfd} of -40, -20, -5, and 5°C. **e**) Maximum stress retention rates of sponges prepared at T_{pfd} of 40, -20, -5, 0, and 5°C after 100 times stress-strain cycles. **f)** Hysteresis loops of stress-strain curves sponges prepared at T_{pfd} of 40, -20, -5, 0, and 5°C. Values and error bars represent the mean and standard deviation (n = 3 independent samples), P > 0.05 (ns), **P < 0.01, ***P < 0.001, ****P < 0.0001 (one-way ANOVA to compare multiple groups or two groups).

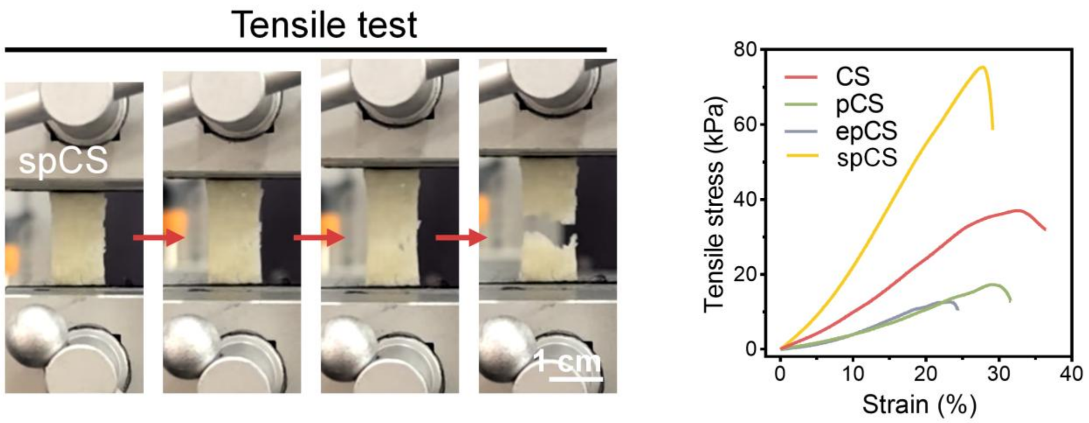

Figure S15. Tensile test of CS, pCS, spCS, and epCS.

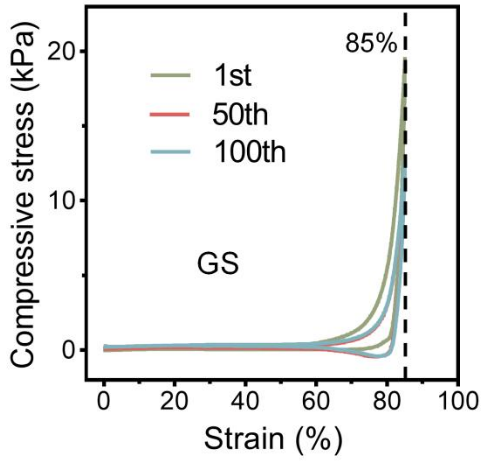

Figure S16. Stress-strain cyclic curves of gelatin sponges (GS).

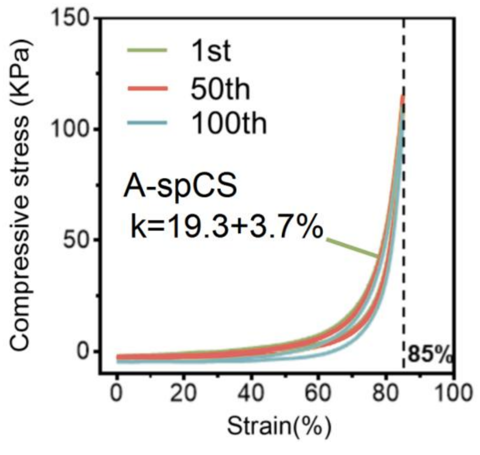

Figure S17. Stress-strain cyclic curves of A-spCS ("k" represents the hysteresis in the first cycle).

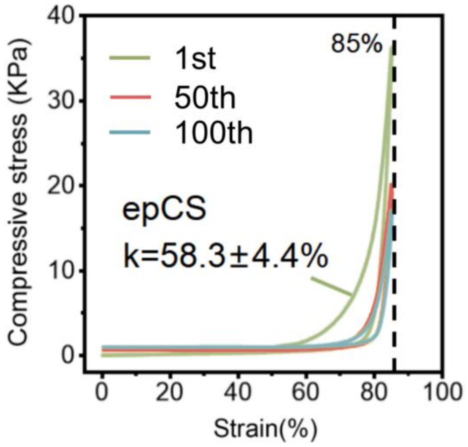

Figure S18. Stress-strain cyclic curves of epCS ("k" represents the hysteresis in the first cycle).

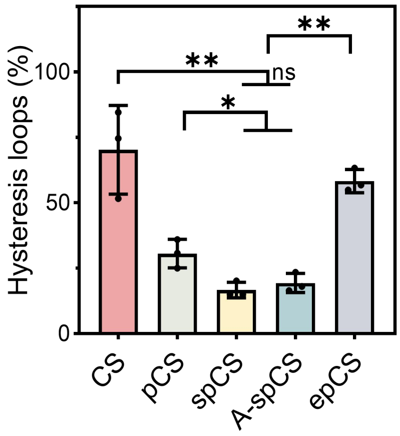

Figure S19. Hysteresis loops of stress-strain curves of CS, pCS, spCS, A-spCS, and epCS. Values and error bars represent the mean and standard deviation (n = 3 independent samples), P > 0.05 (ns), **P < 0.01, ***P < 0.001, ****P < 0.0001 (one-way ANOVA to compare multiple groups or two groups).

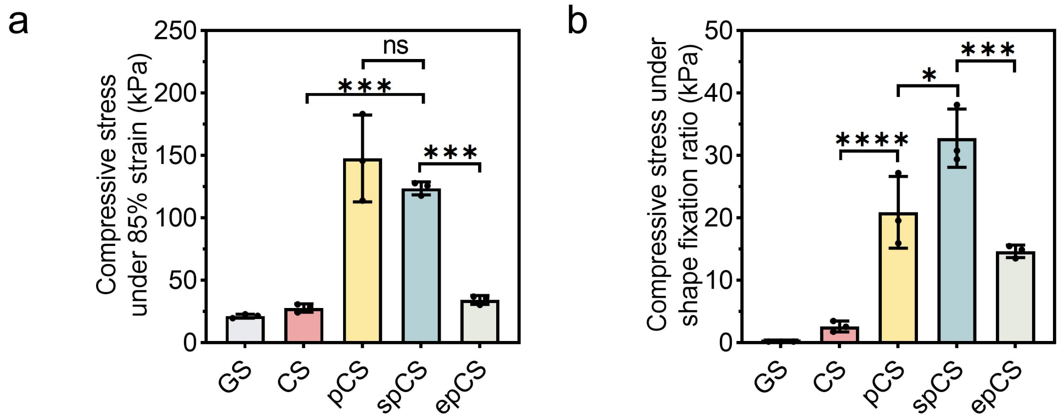

Figure S20. Compressive stress under different strain of gelatin sponge, CS, pCS, spCS, epCS. **a)** Compressive stress under 85% strain of gelatin sponge, CS, pCS, spCS, and epCS. **b)** Compressive stress under shape fixation state of gelatin sponge, CS, pCS, spCS, and epCS. Values and error bars represent the mean and standard deviation (n = 3 independent samples), P > 0.05 (ns), **P < 0.01, ***P < 0.001, ****P < 0.0001 (one-way ANOVA to compare multiple groups or two groups).

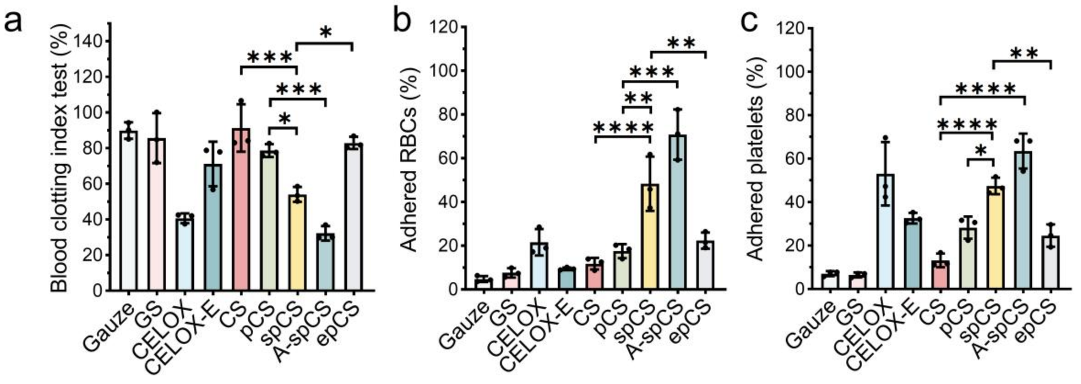

Figure S21. Coagulation effect of various hemostatic materials and blood cell adhesion ability with . **a)** The Blood Clotting Index (BCI) of the hemostatic material tested at three times the normal blood volume for 10 minutes. **b, c)** Percentage of red blood cells and platelets adhered on different hemostatic materials at three times the normal blood volume. Values and error bars represent the mean and standard deviation (n = 3 independent samples), P > 0.05 (ns), **P < 0.01, ***P < 0.001, ****P < 0.0001 (one-way ANOVA to compare multiple groups or two groups).

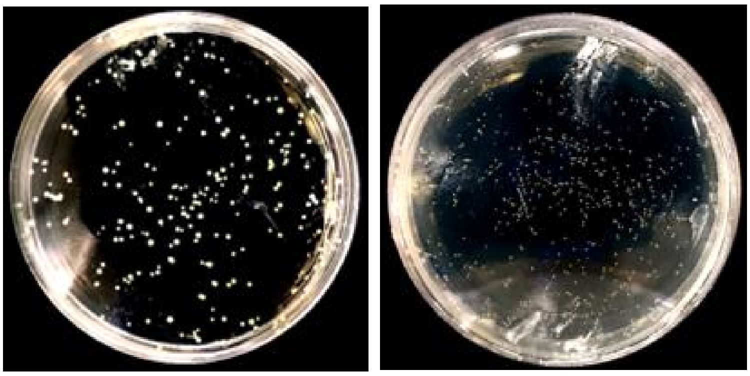

E. coli *S. aureus*

Figure S22. The remaining colonies of *Escherichia coli (E. coli)* and *Staphylococcus aureus (S. aureus)* after 2 h co-incubation with gauze group.

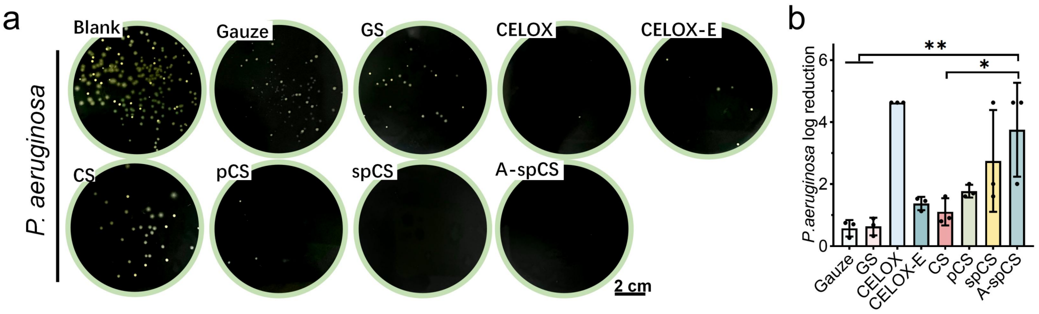

Figure S23. In vitro anti-*Pseudomonas aeruginosa* (*P. aeruginosa*) properties of various hemostatic materials. **a)** Photographs of colonies of *P. aeruginosa* grown on LB agar plates after contact with blank, gauze, GS, CELOX, CELOX-E, CS, pCS, spCS, and A-spCS. **b)** Corresponding statistical results of colony counts of *P. aeruginosa*. Values and error bars represent the mean and standard deviation (n = 3 independent samples), P > 0.05 (ns), **P < 0.01, ***P < 0.001, ****P < 0.0001 (one-way ANOVA to compare multiple groups or two groups).

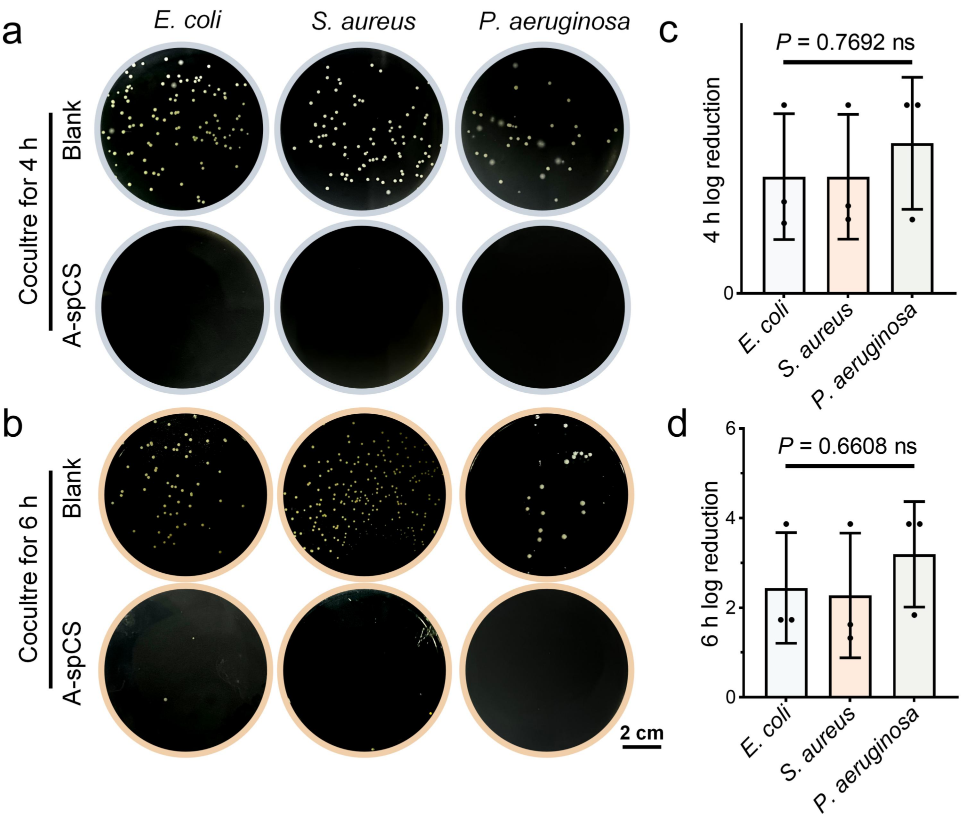

Figure S24. The evaluation of the antimicrobial efficacy of A-spCS after co-incubation for 4 and 6 hours. **a)** Photographs of colonies of *E. coli*, *S. aureus*, and *P. aeruginosa* grown on LB agar plates after contact with blank and A-spCS for 4 h **b)** and 6 h. **c)** Corresponding statistical results of colony counts for 4 h **d)** and 6 h. Values and error bars represent the mean and standard deviation (n = 3 independent samples), P > 0.05 (ns), **P < 0.01, ***P < 0.001, ****P < 0.0001 (one-way ANOVA to compare multiple groups or two groups).

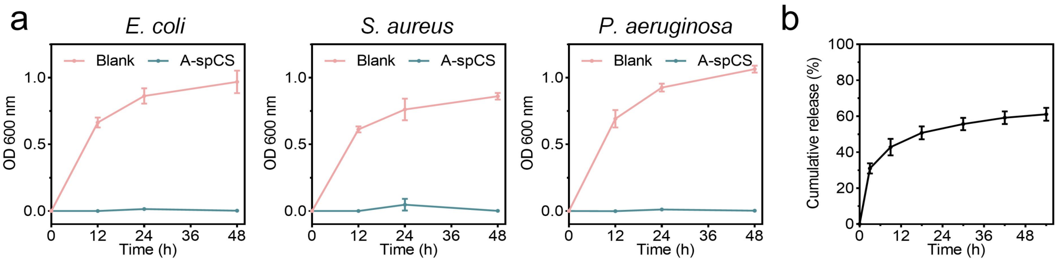

Figure S25. The evaluation of the antimicrobial efficacy of penicillin G-loaded chitosan sponge for 12, 24, and 48 hours. **a**) Absorbance of LB after incubation of *E. coli*, *S. aureus*, and *P. aeruginosa* with penicillin-loaded chitosan sponge for 12, 24, and 48 hours. **b**) Penicillin G sustained release curve. Values and error bars represent the mean and standard deviation (n = 3 independent samples).

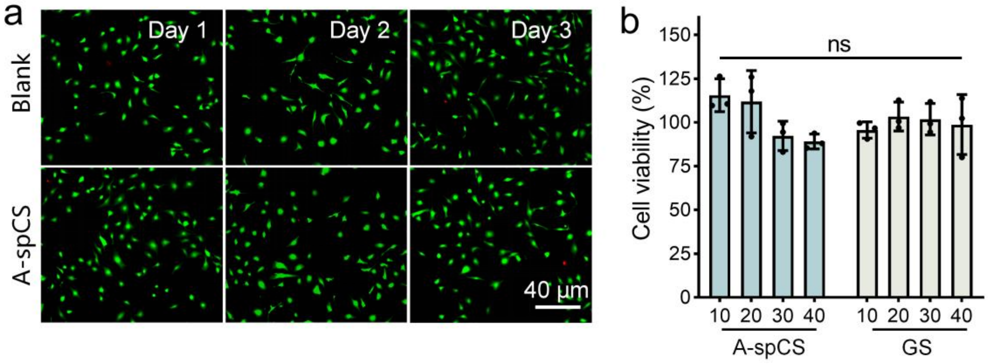

Figure S26. Cell viability test of LX-2 hepatic stellate cell. **a**) Fluorescence microscopy images of live-dead staining of LX-2 hepatic stellate cells after 1, 2, and 3 days of culture in A-spCS extract (n=3). **b**) The CCK-8 tests revealed the cell viability of LX-2 hepatic stellate cells cultured in different mass fractions (10, 20, 30, 40 mg/mL) of material extracts. Values and error bars represent the mean and standard deviation (n = 3 independent samples).

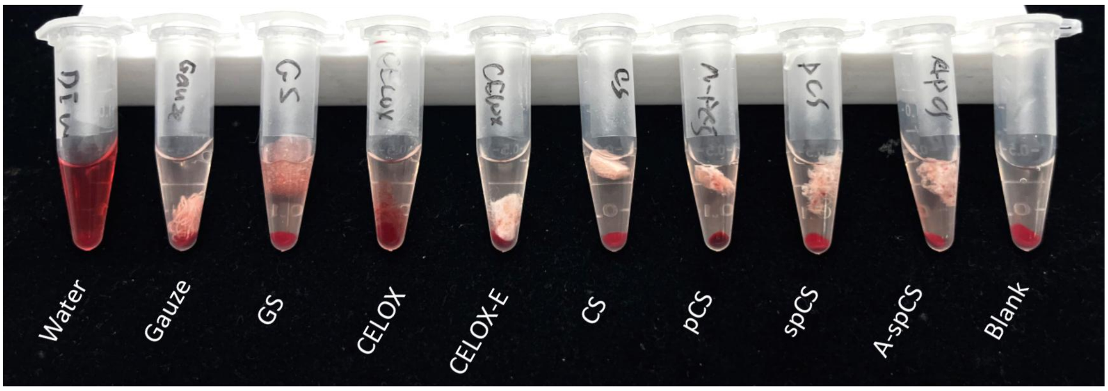

Figure S27. Photo of hemolysis assay of water, PBS, gauze, GS, CELOX, CELOX-E, CS, pCS, spCS, and A-spCS.

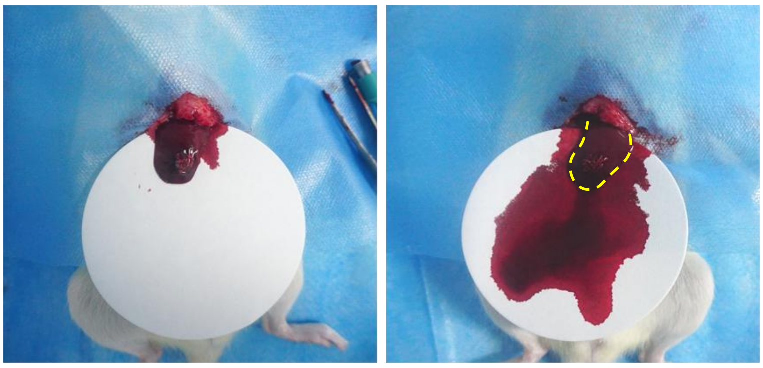

0 s 240 s

Figure S28. Photo of hemostasis in rat liver injury model in gauze group.

Hemostats	Xstat[1]	SHHS-CHS[2]	CMN-Cu[3]	TRAP/SP[4]	OBC-PDA/ PDA-MMT$_3$ /Ag[5]	A-spCS
Shape-recovery Time (blood)	25 s			10 s		4 s
Hemostatic time		55.50±7.00 s	47 s	33±2 s	32±4 s	12.67±12.5 s

Figure S29. Comparison of shape-recovery time and hemostatic time of liver non-compressible wound model between the A-spCS and reported hemostats.

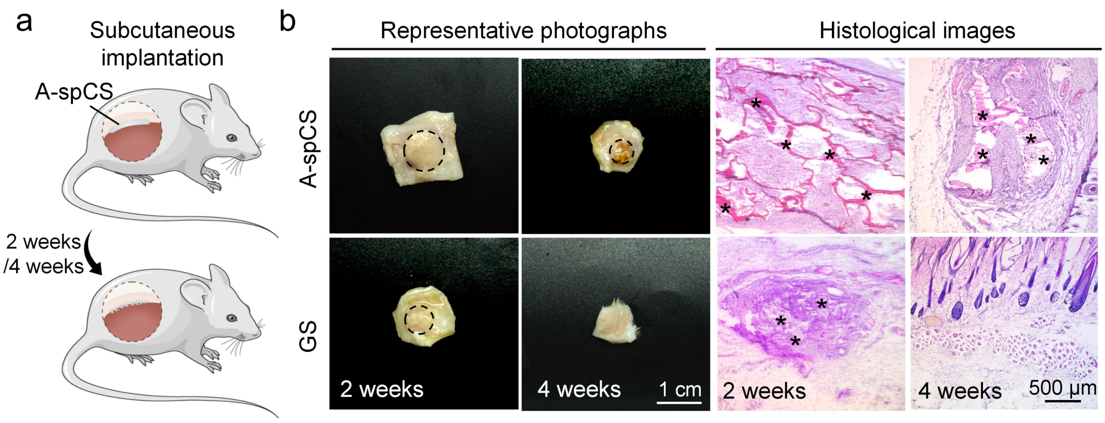

Figure S30. A-spCS and GS in vivo degradation experiment. **a**) Schematic diagram of in vivo explanation. **b**) Representative photos and H&E staining pictures of degradation of A-spCS and GS.

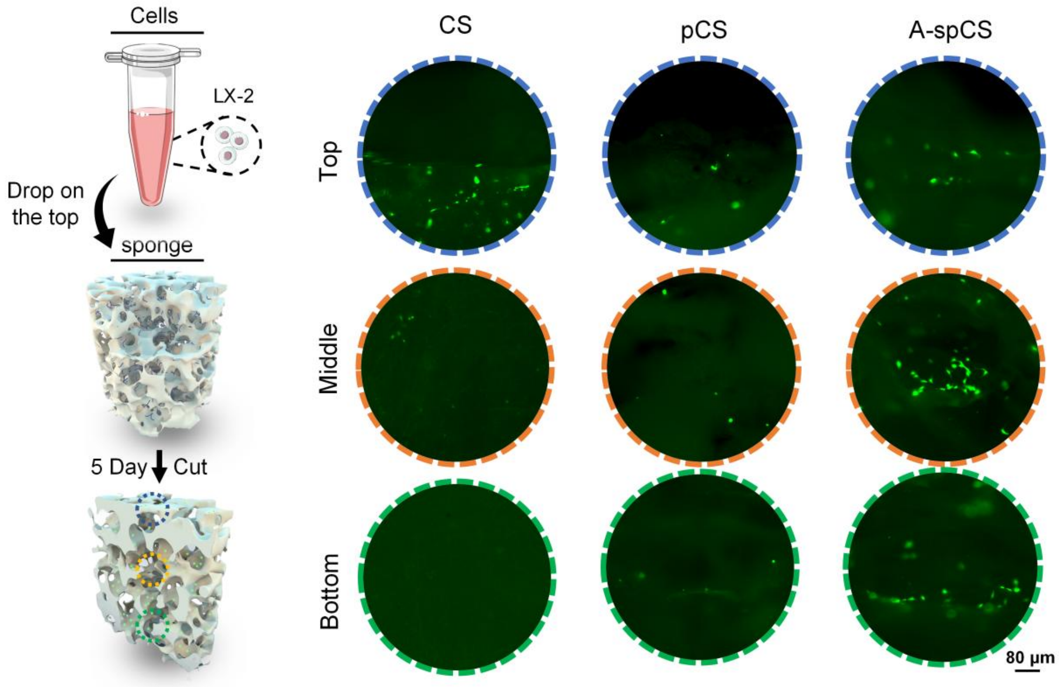

Figure S31. In vitro cell migration experiments of LX-2 within CS, pCS, and A-spCS.

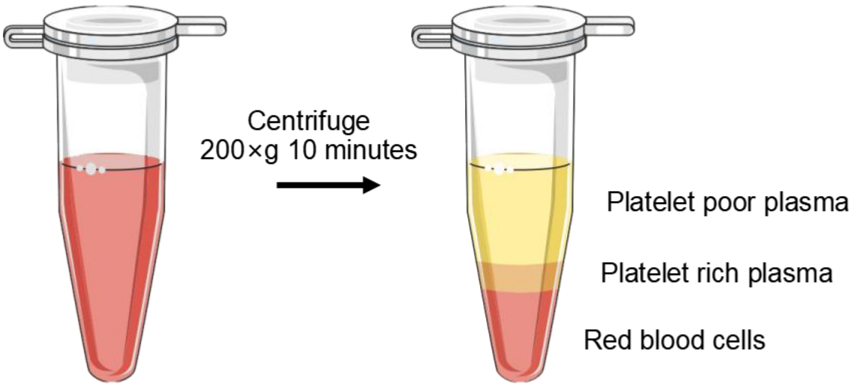

Figure S32. Schematic for preparing red blood cells (RBCs) and platelet-rich plasma (PRP).

Supplementary References

1. Mueller, G. R. *et al.* A novel sponge-based wound stasis dressing to treat lethal noncompressible hemorrhage. *Journal of Trauma and Acute Care Surgery* **73**, S134-S139 (2012).

2. Hu, Z. *et al.* A super hydrophilic and high strength chitosan hemostatic sponge prepared by freeze-drying and alkali treatment for rapid hemostasis. *Materials Today Communications*, 108855 (2024).

3. Huang, Y. *et al.* MXene-NH2/chitosan hemostatic sponges for rapid wound healing. *International Journal of Biological Macromolecules* **260**, 129489 (2024).

4. Yang, X. *et al.* Peptide-immobilized starch/PEG sponge with rapid shape recovery and dual-function for both uncontrolled and noncompressible hemorrhage. *Acta Biomaterialia* **99**, 220-235 (2019).

5. Cao, S., Yang, Y., Zhang, S., Liu, K. & Chen, J. Multifunctional dopamine modification of green antibacterial hemostatic sponge. *Materials Science and Engineering: C* **127**, 112227 (2021).
