# Supplementary material for: Superporous sponge prepared by secondary network compaction with enhanced permeability and mechanical properties for non-compressible hemostasis in pigs
Source: Nat Commun. 2024 Jun 27;15:5460. doi: 10.1038/s41467-024-49578-2 (PMC11211411; doi:10.1038/s41467-024-49578-2)
Supplement: Supplementary file 3 — Description Of Additional Supplementary File [file 41467_2024_49578_MOESM3_ESM.pdf]

**Description of Additional supplementary file**

**Movie S1.** Video of micro-CT scanning of the CS.

**Movie S2.** Video of micro-CT scanning of the pCS.

**Movie S3**. Video of micro-CT scanning of the spCS.

**Movie S4**. Video of micro-CT scanning of the A-spCS.

**Movie S5**. Video of micro-CT scanning of the epCS.

**Movie S6.** Video of water-triggered shape recovery of the CS.

**Movie S7.** Video of water-triggered shape recovery of the pCS.

**Movie S8**. Video of water-triggered shape recovery of the A-spCS.

**Movie S9.** Video of water-triggered shape recovery of the epCS.

**Movie S10**. Video of blood-triggered shape recovery of the CS.

**Movie S11**. Video of blood-triggered shape recovery of the pCS.

**Movie S12.** Video of blood-triggered shape recovery of the A-spCS.

Movie S13. Video of blood-triggered shape recovery of the epCS.

**Movie S14**. Video of hemostasis of the A-spCS in rat liver perforation wound model.

**Movie S15**. Video of hemostasis of the A-spCS in mini pig spleen perforation wound model.

**Movie S16.** Video of hemostasis of the blank group in mini pig spleen perforation wound model.
